# Gut microbial metabolism of amino acids and its impact on enteric infections

**DOI:** 10.1128/iai.00298-26

**Published:** 2026-10-08

**Authors:** Kanchan Jaswal, Judith Behnsen

**Affiliations:** 1 Department of Microbiology and Immunology, University of Illinois Chicago 200799, Chicago, Illinois, USA; University of Pittsburgh, Pittsburgh, Pennsylvania, USA

**Keywords:** gut microbiota, bacteria, fungi, protists, enteric pathogens, nutrient competition, amino acid metabolism

## Abstract

Amino acids are the fundamental building blocks of proteins. Dietary proteins and endogenous protein turnover are the main sources of amino acids in the body. Over the past decade, our understanding of the role of the gut microbiota in amino acid metabolism has expanded considerably. The gut microbiota metabolizes dietary amino acids, regulates intestinal amino acid bioavailability, and maintains host nutrient homeostasis. Besides nutrient regulation, emerging evidence has revealed that amino acid availability in the gut also plays a crucial role in modulating enteric infections. Amino acids can influence infection outcomes both directly, by affecting the virulence of enteric pathogens, and indirectly, by shaping host immune responses. While most studies have focused on bacterial metabolism of amino acids, recent findings highlight important contributions from non-bacterial members of the gut microbiota, including fungi and protists. Both fungi and protists can shape amino acid availability in the gut and, consequently, infection outcomes. In this review, we integrate current knowledge on how diet and amino acid metabolism, mediated by both bacterial and non-bacterial communities, collectively influence enteric infection dynamics.

## INTRODUCTION

A balanced human diet comprises carbohydrates, fats, and proteins, along with vitamins and minerals, which are required to sustain physiological functions. In many Western diets, protein often takes center stage, with recommendations typically falling between 0.8 and 1.5 g/kg of body weight each day. However, what happens to that protein after we eat it is less straightforward than it might seem. This review will first follow proteins through the digestive tract and then examine current knowledge on amino acid availability in the gut. This will set the basis to understand the role of the microbiota in shaping amino acid metabolism, and how these dynamics influence enteric infections, as discussed in detail in this review.

## THE JOURNEY OF PROTEINS THROUGH THE GUT

**Fig 1:**
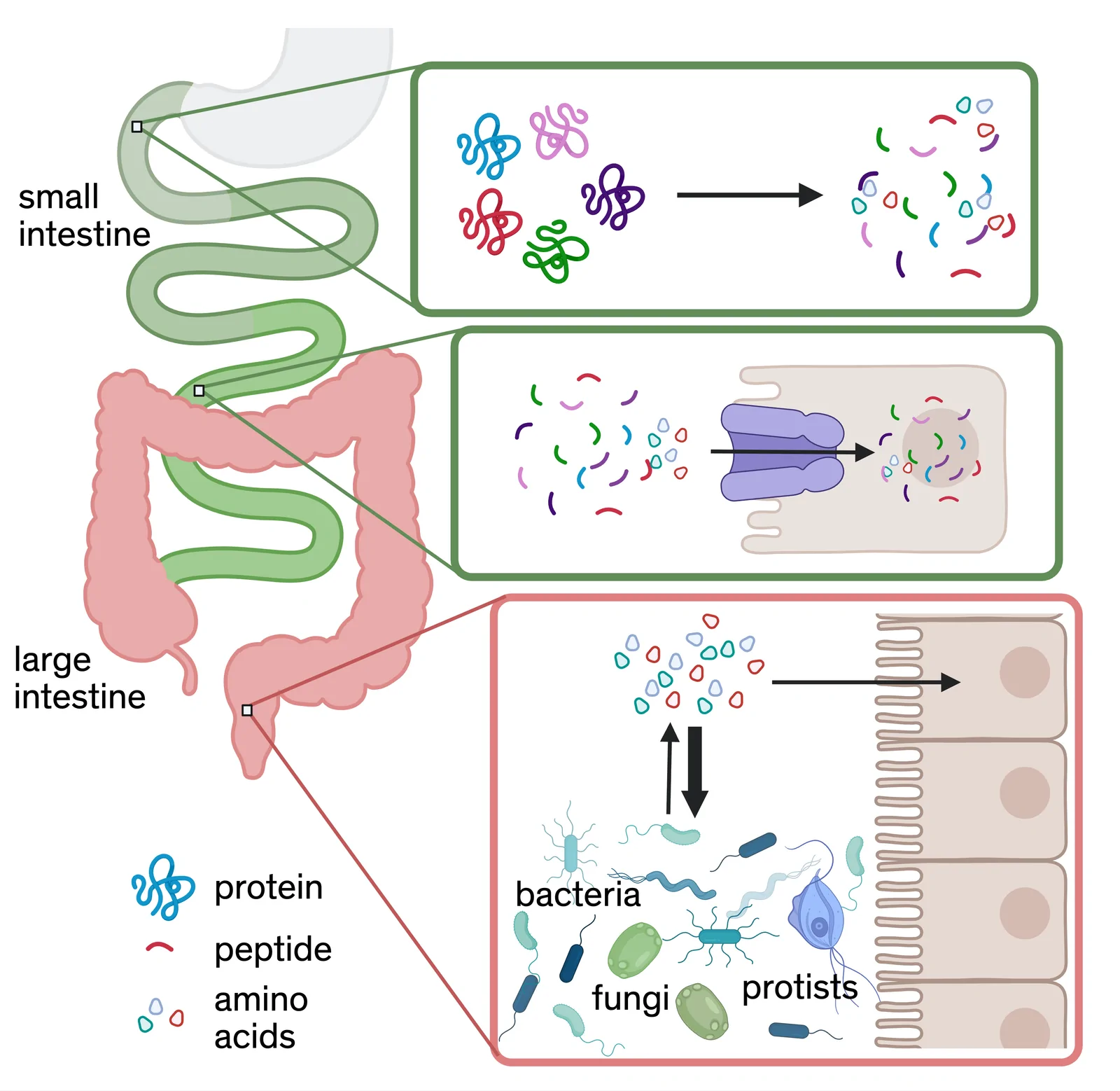
Proteins are broken down into oligopeptides and free amino acids through denaturation and enzymatic hydrolysis and are then primarily absorbed in the small intestine using amino acid-specific transporters. However, a significant amount of the amino acids reaches the large intestine, where they are primarily utilized by the gut microbiota. The gut microbiota further converts them into metabolites that are used by the host. Some members of the gut microbiota, such as fungi, also have the capacity to synthesize amino acids and release them into the intestinal environment.

Proteins are long chains of amino acids, often described as the “building blocks” of the body. As food moves through the digestive tract, proteins are broken down into oligopeptides and free amino acids via denaturation and enzymatic hydrolysis (Fig. 1). Amino acids are absorbed mainly in the small intestine (1) (Fig. 1) and reused by the body to build new proteins, from muscle fibers to enzymes and signaling molecules. However, a small fraction of dietary proteins and amino acids remain undigested or incompletely digested and escape absorption in the small intestine. It has been estimated that approximately 1.5–5 g of nitrogen is transferred through the ileo-cecal junction daily in humans (2–4). The majority of this nitrogen is present as proteins and, to a much lesser extent, peptides, amino acids, urea, ammonia, nitrate, and other compounds (5). Peptides, in fact, are generally absorbed more rapidly than free amino acids in the small intestine. Importantly, the nitrogenous material reaching the distal small intestine is derived not only from dietary sources but also from endogenous sources, including digestive secretion, exfoliated epithelial cells, and mucus (4). Approximately 40% of the nitrogenous material is dietary, whereas ~60% is endogenous (3).

The continuous flow of dietary and host-derived nitrogen into the large intestine creates a nutrient-rich environment that is not fully utilized by the host and instead becomes available to the resident gut microbiota (Fig. 1). Amino acid availability in the large intestine is further shaped by limited host absorption in the colon despite the presence of peptide transporters such as PepT1 (6). The rapid turnover of the gut microbiota, along with the loss of nutrients through defecation, creates a system in the colon that necessitates a continuous supply of nutrients, largely provided by the host (4, 7). Therefore, unlike the small intestine, where host absorption dominates, the large intestine functions as a site of intense microbial metabolism.

The gut thus represents a shared metabolic environment in which the host and microbiota both compete for and exchange nutrients. The resident gut microbiota is a complex, multispecies ecosystem that includes bacteria, fungi, viruses, archaea, and protists (8). Gut microbial communities may modulate host amino acid availability through several mechanisms. For instance, gut bacterial colonization can affect intestinal protein-digestion enzymes, such as trypsin (9). Gut microbiota colonization can also modify intestinal permeability by modulating gut amino acids that affect tight junctions (10, 11). Additionally, gut microbes directly metabolize amino acids in the intestine, as discussed in the next section.

## AMINO ACID METABOLISM IN THE GUT

Metabolites in the gut are frequently exchanged between the host and the microbiota, with diet serving as a major driving force shaping this interaction. The gut microbiota can produce metabolites through both anabolic and catabolic processes: resident microbes utilize dietary or host-derived nutrients to biosynthesize macro- and micronutrients while also converting these substrates into various bacterial metabolites via fermentation and other pathways. Among the many metabolites exchanged in the gut, short-chain fatty acids (SCFAs; acetate, propionate, and butyrate) are the most extensively studied. They provide a clear example of how diet influences microbial metabolism. However, they represent only one part of the metabolic interactions in the gut and highlight how little is known about amino acid metabolism. SCFAs are primarily generated by microbial fermentation of dietary substrates by *Clostridium* and *Bacteroides* species (12). In addition, while bacteria from the genera *Lactobacillus* and *Bifidobacterium* do not produce SCFAs themselves, they support SCFA production through metabolic cross-feeding. This breakdown of dietary fiber provides the substrate (oligosaccharides, lactate, and acetate) for other SCFA-producing bacteria (13, 14). Dietary composition thus plays a major role in shaping the gut microbiota, selecting for microbes that preferentially digest specific nutrients, and can induce rapid, though often transient, shifts in gut microbial composition and diversity (15). In contrast to SCFA metabolism, amino acid metabolism in the gut remains less understood. The following section focuses on amino acid metabolism carried out by both bacterial and non-bacterial components of the gut microbiome.

### Bacterial amino acid metabolism

Amino acids are primarily absorbed in the proximal site of the intestine. Consequently, amino acids are much less abundant in the lower gastrointestinal tract. Yet, this relationship is more complex than it appears. If amino acid levels were simply a function of diet and host uptake, then levels measured by targeted metabolomics would be similar in germ-free mice compared to mice with a gut microbiome. However, mice colonized with microbes have significantly lower luminal amino acid levels than germ-free mice. The differences in amino acid pools between germ-free mice and microbe-colonized mice become more pronounced the denser the microbial population gets. The differences in amino acid concentration are thus most pronounced in the lower gastrointestinal tract, which has a denser population of microbiota than the upper gastrointestinal tract (16). The gut microbiota is therefore a major consumer of luminal amino acids in the distal gut. In the proximal gut, the host remains the main consumer of amino acids. However, the host can also be a producer of amino acids, e.g., via endogenous secretions in the colon. This can be inferred from experiments with germ-free mice, where amino acid pools in the colon are high even though the host efficiently absorbs amino acids in the small intestine (4, 7). There are thus additional sources of amino acids besides diet in the gut.

While gut bacterial commensals are major consumers of amino acids, they also possess the metabolic capacity to biosynthesize them. This raises an important question: does *de novo* amino acid biosynthesis by the microbiota contribute to amino acid levels in the gut? Dissecting whether a given metabolite is host- or microbiota-derived using metabolomics remains challenging, as many metabolic pathways are conserved between microbes and the host. However, integrative approaches combining metabolomics with *in silico* metabolic modeling have provided insights into this complexity. Interestingly, more than half of the metabolic reactions within the gut environment are microbiota-dependent, with amino acid metabolism representing one of the largest functional categories (17).

The capacity for amino acid biosynthesis, however, varies across microbial taxa. Some taxa, such as *Proteobacteria*, possess extensive amino acid metabolic capabilities, whereas others, such as *Lactobacillus*, rely heavily on environmental sources (17). Similarly, several pathogens, such as *Campylobacter jejuni*, *Helicobacter pylori*, *Enterococcus faecalis*, and *Streptococcus agalactiae*, have lost key amino acid biosynthetic pathways (18). These pathogens survive but generally do not replicate outside of their hosts and are thus specifically adapted to this environment. This has led to the hypothesis that some commensal and pathogenic gut bacteria have lost amino acid biosynthetic pathways due to their consistent availability in the gut environment (19), suggesting adaptation to nutrient-rich niches. Indeed, even bacteria that retain biosynthetic capacity often rely heavily on the consumption of exogenous amino acids when available. Isotope-labeling studies in the pig ileum showed that luminal amino acids derived from dietary and endogenous host sources constituted the primary substrates for bacterial protein synthesis, whereas *de novo* bacterial amino acid biosynthesis plays a relatively minor role (20). Nevertheless, there are specific examples indicating that the gut microbiota can still serve as a source of both essential and non-essential amino acids to the host. For example, it has been estimated that up to 60% of skeletal muscle valine in mice is derived from the gut microbiota (21). In addition, gut microbes indirectly contribute to increased amino acid availability. They produce proteases such as trypsin that facilitate the breakdown of proteins resistant to host enzymatic digestion, thereby further modulating amino acid availability in the gut (9).

### Amino acid metabolism by non-bacterial members

Bacteria vastly outnumber other members of the gut microbiome, and historically, more emphasis has been placed on their contributions to the gut environment than on other members. As a result, studies that have examined amino acid metabolism in the gut have largely focused on bacteria or the microbiome in general. Consequently, these studies have failed to resolve the contributions of other members of the gut microbiome. Protists, fungi, and archaea also contribute directly to amino acid metabolism in the gut or have the capabilities to do so. Among archaea, *Methanobrevibacter* is the most prevalent genus, present in approximately 30% of the human population. *Methanobrevibacter smithii* encodes transporters for aspartate, arginine, glutamate, glutamine, as well as histidine, and has been shown to consume several amino acids *in vitro* (22). In addition, by consuming hydrogen and other fermentation byproducts of amino acid degradation, archaea indirectly influence amino acid turnover in the gut. However, their contribution to the overall gut amino acid metabolome has not been directly examined.

#### Protists amino acid metabolism

Protists represent another understudied group present in the gut. Commensal protists are present in healthy mammalian guts and can strongly influence intestinal immunity in mice (23–26). Parabasalid protists of the genus *Tritrichomonas* are murine commensals and are widespread in laboratory mice (26–28). In general, protists are known to rely on external sources of amino acids for their growth (29–32). The protist *Tritrichomonas musculus* (*Tmu*), e.g., utilizes arginine as an energy source (33). *In vitro*, *Tmu* has been shown to convert arginine into citrulline and ornithine, possibly by the arginine dihydrolase pathway, and release these metabolites into the surrounding media. *In vivo*, metabolomics data of the mouse cecum showed that the presence of *Tmu* is associated with amino acid richness, with a reduction in arginine levels and an increase in ornithine, citrulline, putrescine, and 5-aminovalerate (33). Interestingly, the presence of *Tmu* also increased the expression of several bacterial arginine dihydrolase genes, including a *Lactobacillales* arginine deiminase pathway and *Helicobacter* ornithine transcarbamylase (34). These findings suggest that gut bacteria may compete for amino acids, such as arginine and tryptophan-derived indole, in the presence of protists.

#### Fungal amino acid metabolism

Fungal members of the gut microbiota also include species that preferentially utilize amino acids as a nitrogen source and possess the ability to convert these amino acids into carbohydrates. The yeast *Candida albicans* is a human commensal present in more than 60% of the population and is the most frequently isolated fungus from the gut of humans (35, 36). In humans, *C. albicans* abundance is negatively associated with a protein-rich diet (37). In contrast, *in vitro* studies report a positive correlation between *C. albicans* growth and amino acid uptake (38, 39). This apparent discrepancy suggests that, although *C. albicans* can efficiently use amino acids as both carbon and nitrogen sources to support growth, its abundance in the gut is not determined by nutrient availability alone. The complex environment of the gut, with the presence of other microbiota members, might influence the actual abundance of this fungus.

Nitrogenous compounds are toxic to yeast cells and are usually disposed of as amino acids to remove excess or potentially toxic levels of intracellular nitrogen. Toxic levels of nitrogen in a yeast cell can arise under two conditions: (i) when the cell takes up excessive amino acids (40) or peptides (41) and (ii) when it overproduces amino acids (42). In condition i, nitrogen-rich environments can drive imbalanced nutrient uptake by gut fungi. These conditions trigger regulatory responses via nitrogen catabolite repression-sensitive pathways, which promote the secretion of excess nitrogen in the form of amino acids. In condition ii, dysregulated amino acid biosynthesis leads to secretion. The role of dysregulated biosynthesis is supported by experiments showing that growth in amino acid-depleted media or mutations affecting uptake systems lead to altered amino acid secretion (40, 42). Yeast cells can secrete amino acids even in the absence of external supplementation, although typically at low levels (43). Classical genetic evidence further supports this concept: arginine auxotrophs can be rescued when grown in proximity to mutants defective in arginine uptake, indicating leakage of intracellular amino acid pools from transporter-impaired strains (42).

The released amino acids by the yeast are cross-fed to neighboring microbial species. For example, *Saccharomyces cerevisiae* releases amino acids such as glutamine and serine, which support the growth of lactic acid bacteria (LAB), including *Lactococcus lactis* and *Lactobacillus plantarum* (44). Interestingly, LAB are frequently found alongside *Saccharomyces cerevisiae* in fermented foods and beverages, including kefir, kimchi, wine, sourdough, and cocoa (45–49). These foods also serve as sources of LAB inoculation in the gut microbiome (50). Notably, LABs are themselves auxotrophic for amino acids and usually rely on environmental pools (17) or, as these findings suggest, on neighboring microbes for amino acid availability.

This modulation of fungal amino acid biosynthesis is also observed during cross-kingdom interactions. *Salmonella enterica* serovar Typhimurium (*Salmonella*) induces a targeted increase in arginine biosynthesis in *Candida albicans* via the type 3 secretion system-1 (T3SS-1). This increased arginine is then released outside, where it is used by *Salmonella* and the host (51). Another example of pathogen-mediated induction of amino acid biosynthesis in *C. albicans* is observed with *Serratia marcescens. S. marcescens* uses its type 6 secretion system (T6SS) effector Tfe2 to cause global changes in *C. albicans* metabolism, likely due to hyperactivation of the TORC1 kinase (52). The changes include a decreased abundance of amino acid transporters, including arginine-specific permeases that import arginine. Contrary to arginine transporters, proteins involved in arginine biosynthesis were more highly abundant, indicating a compensatory upregulation of arginine biosynthesis (53). Higher arginine levels were confirmed in *S. cerevisiae*, where expression of Tfe2 significantly increased intracellular arginine. However, whether this arginine is released into the surrounding environment was not determined. These observations collectively suggest that disruption of amino acid homeostasis in yeast, whether by direct modulation or through metabolic imbalance, can drive extracellular release.

Interestingly, yeasts do not seem to use direct efflux via transporters in the plasma membrane for amino acid release. Instead, vesicle-mediated export has been proposed as a key pathway for the release of amino acids to the extracellular environment. The vesicular transporter Aqr1 has been implicated in the efflux of multiple amino acids. In a proposed model, excess cytosolic amino acids are sequestered into intracellular vesicles that subsequently fuse with the plasma membrane and release their contents into the extracellular environment (40). This process is conceptually analogous to neurotransmitter exocytosis in neurons and highlights a regulated, rather than passive, mode of amino acid export in fungi (40, 42).

The contribution of fungi to the metabolic environment of the gut is currently largely unknown. It might be minimal, as suggested in reference 54. The fecal metabolome of germ-free mice colonized with defined fungal species only minimally changed, with amino acid asparagine being one of the few metabolites that was more abundant (54). However, it would be interesting to determine whether fungi serve as a source of amino acids in the gut. Under specific conditions, such as the presence of *Salmonella*, commensal fungi can serve as a significant source of amino acids (51).

## GUT MICROBIAL AMINO ACID METABOLISM IN HEALTH AND DISEASE

Despite being restricted to the gut lumen, the gut microbiota influences not only local but also systemic amino acid levels. Colonization studies using highly effective amino acid metabolizers, such as *Clostridium sporogenes*, *Bacteroides ovatus*, and *Clostridium senegalense*, demonstrated the ability of gut microbiota to modulate the amino acid landscape not only in the gut and stool but also in peripheral tissues. The concentrations of most amino acids, such as histidine, lysine, proline, and tryptophan, were significantly decreased in the serum samples of colonized mice (16). Therefore, gut microbiota amino acid utilization alters blood amino acid levels, which can directly influence physiological functions in the body.

In line with these findings, alterations in microbiota-driven amino acid metabolism are increasingly linked to disease. In cancer, changes in the gut microbiome are strongly associated with responses and efficacy of immunotherapy (55–57). Microbial consumption of amino acids such as asparagine by *Bacteroides* depletes the local availability of asparagine to T cells. This decrease in gut asparagine downregulates the expression of the asparagine transporter (SLC1A5) in T cells. This impairs T cells’ stem-like properties and effector functions, which are required to fight tumors, thereby affecting tumor progression (58). Similarly, metabolic disorders such as type 2 diabetes are associated with disrupted amino acid levels. Gut microbiota genes involved in branched-chain amino acid and tryptophan metabolism affect host glucose homeostasis partly through peripheral serotonin pathways (16). Because these functions are microbiota dependent, changes in gut microbial composition and stability can disrupt amino acid metabolism and worsen metabolic disease outcomes.

In addition to amino acids themselves, breakdown products of amino acids can have significant effects on the host. Microbial utilization of intestinal amino acids produces a wide range of metabolites with both beneficial and detrimental effects. For example, deamination, decarboxylation, or desulfurization of amino acids can generate compounds such as ammonia, hydrogen sulfide, and p-cresol, which at high concentrations impair epithelial functions, disrupt mitochondrial respiration, and promote inflammation (19). However, amino acid fermentation also yields beneficial metabolites, including SCFAs and branched-chain fatty acids, which support host energy metabolism and barrier integrity (59, 60). Microbial metabolism of amino acids also produces bioactive compounds such as polyamines, histamine, and tryptophan-derived indoles, many of which contribute to gut homeostasis and host signaling (19, 61). Certain members of the *Fusobacterium* genus and *Clostridium* species, such as *Clostridium tetani* and *Clostridium tetanomorphum*, can synthesize SCFAs, particularly butyrate, via amino acid fermentation (62). This process generates ammonia as a byproduct, which has been increasingly associated with colorectal cancer. Consistent with this, increased abundances of *Fusobacterium nucleatum* have been reported in patients with colorectal cancer compared to healthy individuals, leading to its proposal as a potential microbial biomarker for disease progression (62, 63). However, it remains unclear whether such microbial shifts are a cause or consequence of cancer (64).

In addition to metabolic functions within the gut, amino acid-derived microbial products also contribute to gut–brain communication. The gut microbiota can influence neurotransmitter levels via two different means: by modulating host biosynthetic pathways and by directly producing neurotransmitters such as GABA, dopamine, and tryptamine. For example, tryptophan decarboxylases of *Clostridium sporogenes* and *Ruminococcus gnavus* can catalyze the conversion of tryptophan to tryptamine (65). Moreover, *Lactobacillus* and *Enterococcus* play a role in dopamine synthesis (66, 67), while glutamic acid decarboxylases of *Bacteroides*, *Parabacteroides*, and *Escherichia* show clear transcriptional activation in human stool samples (68). The gut microbiota also converts dietary tyrosine to 4-ethylphenol (4EP), which enters the brain and disrupts oligodendrocyte maturation and myelination patterns in the brain. Similarly, p-cresol, a tyrosine-derived metabolite structurally related to 4EP, has been suggested to alter oligodendrocyte function and influence social and depression-like behaviors in mice (69, 70).

## ROLE OF AMINO ACIDS IN ENTERIC INFECTION

One important role of the gut microbiota is protection of the host against enteric infections, a process known as colonization resistance. Colonization resistance is a complex phenomenon, but roughly, the mechanisms can be divided into direct and indirect effects. Direct effects include inhibition or killing of invading pathogens and competition for essential nutrients, whereas indirect effects involve modulation of host immune responses. In an adult gut with a complex microbiome, key nutrients such as carbohydrates, amino acids, and respiratory electron acceptors (e.g., oxygen) are tightly limited due to efficient scavenging by both host tissues and resident microbes. As a result, it has been proposed that competition for a small number of critical nutrients, rather than the production of inhibitory compounds alone, plays a dominant role in determining microbial composition and stability (71, 72). Within this framework, amino acids represent a particularly important resource. Their availability and utilization can influence microbial fitness, shape interspecies competition, and modulate host immunity. In the next section, we will discuss the different mechanisms by which amino acids affect enteric infections.

### Nutrient competition

**Fig 2:**
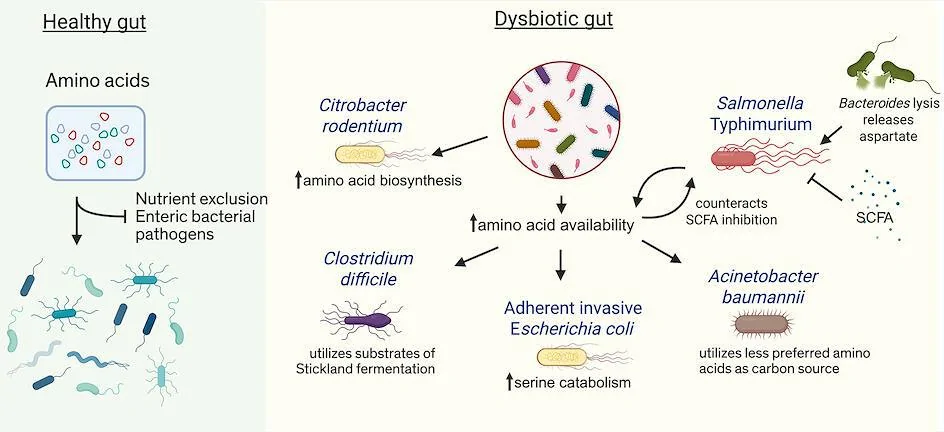
Microbiota utilization of amino acids in the gut creates an amino acid-limited environment, increasing competition for these scarce nutrients. To overcome this limitation, enteric pathogens have evolved diverse metabolic strategies. For example, *Citrobacter rodentium* enhances amino acid biosynthesis during infection, whereas adherent-invasive *Escherichia coli* increases serine utilization. *Acinetobacter baumannii* can exploit non-preferred amino acids, such as ornithine, as alternative carbon sources. In dysbiotic conditions, elevated amino acid availability provides substrates for Stickland fermentation, promoting the growth of *Clostridioides difficile*. Similarly, *Salmonella* can increase amino acid availability to overcome short-chain fatty acid-mediated inhibition. Additionally, the lysis of commensal microbiota releases amino acids such as aspartate, which *Salmonella* utilizes to support intestinal colonization.

Germ-free mice, devoid of any microbes, exhibit significantly higher levels of free amino acids in the distal gut compared to conventionally raised animals. This observation supports the idea that the resident microbiota utilizes amino acids in the large intestine, thereby creating a nutrient-limited environment (16). The introduction of an enteric pathogen to a complex microbiota imposes additional competition for these already limited resources (Fig. 2).

While the microbiota restricts the availability of amino acids, enteric pathogens have evolved strategies to overcome this limitation. For example, *Citrobacter rodentium*, a mouse pathogen and a widely used model for enteropathogenic *Escherichia coli* and enterohemorrhagic *E. coli* (EHEC) infections, induces its amino acid biosynthetic pathways to overcome this colonization resistance (Fig. 2). Interestingly, amino acid biosynthesis was dispensable for *C. rodentium* colonization in germ-free conditions but was required in the presence of complex microbiota (73), highlighting that nutrient competition promotes metabolic flexibility in enteric pathogens. One of the predominant EHEC pathogens, *E. coli* O157:H7, was found to be particularly reliant on proline for colonization. Consequently, EHEC expansion was reduced in the presence of commensal *E. coli* that compete for proline (74).

A similar dependency on amino acids was observed during *Salmonella* infection. In antibiotic-treated mouse models, protein-rich diets promoted pathogen expansion, whereas protein-free diets did not (75). Another study showed how amino acids can counteract colonization resistance during *Salmonella* infection (76). The gut microbiota produces SCFAs, which can be protonated in the lumen of the colon. In protonated form, SCFAs can pass freely through the bacterial membrane, which decreases the intracellular pH and inhibits the growth of pathogens like *Salmonella* (77). To counteract intracellular acidification, *Salmonella* utilizes amino acids. Infection with *Salmonella* leads to decreased host amino acid absorption in the small intestine and, consequently, increased amino acid availability in the cecum, a primary site for *Salmonella* colonization. *Salmonella* takes up the now more abundantly available amino acids, such as lysine and ornithine. Decarboxylation of these amino acids increases the intracellular pH, which enables *Salmonella* to overcome SCFA-induced stress and proliferate (76) (Fig. 2).

Inflammation further enhances pathogen access to amino acids. *Salmonella*-induced inflammation increases the availability of host-derived respiratory electron acceptors, including oxygen, nitrate, tetrathionate, and fumarate, which benefits the pathogen’s growth in the gut (78–81). Host-derived reactive oxygen species induce the lysis of commensal bacteria, releasing amino acids, such as aspartate, into the gut lumen. *Salmonella* utilizes this microbiota-derived aspartate via aspartate-ammonia lyase-dependent fumarate respiration to support growth specifically under inflammatory conditions (82) (Fig. 2). This amino acid-dependent growth advantage is absent in germ-free mice but restored upon colonization with defined microbial communities (75, 82).

Access to amino acids is not only important for gram-negative pathogens but also for gram-positive bacteria, such as *Clostridioides difficile*. Infection with this pathogen commonly follows antibiotic-induced disruption of the microbiota. The increased amino acid availability in a dysbiotic gut environment supports *C. difficile* expansion (83) (Fig. 2). In addition, host inflammation drives the degradation of extracellular matrix components, such as collagen, releasing peptides rich in proline and glycine. These amino acids are the key substrates of Stickland fermentation, an anaerobic process that utilizes amino acids as electron donors and acceptors. In the gut, *C. difficile* increases expression of amino acid transporters and the associated fermentation pathways involved in Stickland fermentation. This upregulation suggests Stickland fermentation pathways are important for *C. difficile* growth in the gut (84, 85). Consistent with this, inhibition of proline utilization, a substrate for Stickland fermentation, impairs gut colonization by *C. difficile*. Dietary restriction of either protein in general or just proline significantly reduces pathogen burden. Importantly, commensal bacteria, particularly members of the class *Clostridia*, can compete for the same amino acid substrates. Thus, infection with *C. difficile* is rare in the context of a mature non-dysbiotic gut environment with high levels of *Clostridia* (86).

There are many other examples that point to the notion that metabolic flexibility is a common feature associated with enteric pathogens. Adherent-invasive *E. coli* (AIEC), for instance, shifts toward L-serine catabolism in the inflamed gut. Although this pathway provides AIEC with a growth advantage in an inflamed environment, this pathway is less relevant in homeostatic conditions. Interestingly, inflammation-induced bloom of AIEC is also reduced by a decrease in dietary amino acids (87). *Acinetobacter baumannii* is another pathogen that shows high metabolic flexibility during gut colonization. *A. baumannii* is primarily associated with lung infections in hospital settings but can asymptomatically colonize the gut, forming a gut reservoir for clinical infection (88–90). Unlike many bacteria, *A. baumannii* uses amino acids as a carbon source and prefers glutamate over other amino acids. It therefore grows well in a dysbiotic gut environment when competition by the microbiota for glutamate is low. However, pathogenic *A. baumannii* is also adapted to non-dysbiotic gut environments where preferred amino acid levels are low. Contrary to non-pathogenic *Acinetobacter* species, it carries a duplication of an arginine succinyltransferase operon, which enables ornithine catabolism. The ability to utilize ornithine as a non-preferred amino acid in the gut environment allows *A. baumannii* to compete with the resident microbiota and maintain gut colonization in mice (91, 92).

### Direct metabolic cue for virulence

Beyond their role in nutrient competition, amino acids have emerged as mediators of inter-microbial interactions as well as direct regulators of virulence in enteric pathogens. For instance, arginine has been shown to directly regulate virulence in the enteric pathogen EHEC via the arginine-sensing transcription regulator ArgR (93).

A fascinating example of an amino acid-mediated interaction can be found between *C. difficile* and enterococci. Enterococci enhance *C. difficile* fitness in the gut by supplying fermentable amino acids, such as leucine and ornithine. Furthermore, amino acid metabolism exerted by enterococci serves as a metabolic cue for *C. difficile* to increase virulence gene expression. Enterococci acquire and metabolize arginine available in the gut lumen and release ornithine. This arginine depletion is sensed by *C. difficile*. Under these nutrient-limiting conditions, an alternate sigma factor, TcdR, induces the expression of toxins TcdA and TcdB, linking amino acid availability to virulence (94). This principle also extends to inter-kingdom interactions. The commensal protist *Tmu* actively metabolizes arginine in the gut, altering the arginine–ornithine balance and thereby influencing the gut’s metabolic environment. These changes, alongside broader immune effects of *Tmu*, such as inhibiting neutrophil recruitment and IL-1β secretion while promoting Th1 cell differentiation and IFN-γ secretion, decrease *C. difficile* virulence (33). Consistent with this, supplementing arginine in drinking water or feeding an ornithine-free diet decreased *C. difficile* infection severity in mice (33, 94).

The role of arginine in inter-kingdom interactions is also evident in fungal–bacterial crosstalk. The commensal yeast *C. albicans* can enhance *Salmonella* virulence by a metabolic feedback loop. In this interaction, *Salmonella* uses its T3SS-1 against *C. albicans* to induce arginine biosynthesis, which is then released into the environment and sensed by *Salmonella* to further increase virulence, particularly by inducing T3SS-1 expression (51). An increase in arginine biosynthesis and its assumed release by *C. albicans* has also been linked to increased virulence in *Actinomyces viscosus*, an oral pathogen involved in root caries (95). It therefore depends on the pathogen whether the presence of arginine will result in increased (i.e., *Salmonella*) or decreased (i.e., *C. difficile*) virulence (33, 51, 94).

Microbiota-derived amino acid metabolites can also exert direct effects on pathogens. For example, valerate, a metabolite derived from amino acid fermentation (including proline metabolism) by the gut microbiota, selectively inhibits the growth of *C. difficile* while sparing commensal bacteria via an unknown mechanism (96). Tryptophan, an essential amino acid obtained from the diet, is extensively metabolized by commensal bacteria into indole-containing compounds that can differentially regulate virulence across different pathogens. In *Salmonella*, indole induces genes associated with efflux-mediated multidrug resistance while repressing T3SS-1 invasion genes (97, 98). In contrast, in EHEC, indole enhances T3SS activity, increasing EspA and EspB secretion and promoting the formation of attaching and effacing lesions on the gut epithelium (99). The gut microbiota also produces D-amino acids, which represent another mechanism the microbiota utilizes to control enteric pathogens. D-tryptophan, e.g., has been shown to inhibit the growth of the enteric pathogen *C. rodentium*. Treatment with D-tryptophan increases the levels of indoleacrylic acids in *C. rodentium*, which are positively correlated with the pathogen’s susceptibility to D-tryptophan. Importantly, an indole-acrylic acid-supplemented diet increased mouse survival after *C. rodentium* infection (100).

### Modulation of the host response

The gut microbiota participates in immunometabolism by producing metabolic products such as SCFAs and bile acid derivatives, which influence immune cell function. In addition to these well-characterized metabolites, amino acids and their derivatives also play a crucial role in shaping host immunity. They can modulate host responses within the gut and thereby indirectly influence susceptibility to enteric infections. In general, amino acids contribute to intestinal homeostasis and repair and maintain immune homeostasis, as discussed in detail in previous reviews (101–105). Dietary cysteine, e.g., enhances intestinal stem cell-mediated regeneration following injury by supporting coenzyme A biosynthesis in intestinal epithelial cells. This promotes the expansion of intraepithelial CD8αβ^+^ T cells and increases interleukin-22 production, thereby promoting epithelial repair (106). Histamine, produced from histidine by *Lactobacillus reuteri*, has also been shown to reduce pro-inflammatory cytokine secretion and decrease colonic inflammation in mice (107). In addition, the presence of amino acids, particularly tryptophan, in the diet enhances the expression of intestinal antimicrobial peptides, maintains gut microbial homeostasis, and reduces susceptibility to intestinal inflammation (108). Luminal D-amino acids can be degraded by the host’s D-amino acid oxidase (DAO), which generates hydrogen peroxide and thus contributes to antimicrobial defense (109). However, certain pathogens, such as *Salmonella*, have evolved mechanisms to evade DAO-mediated killing. *Salmonella*, when engulfed by neutrophils, limits the DAO substrate, D-alanine, by increasing its uptake. This subversion of killing by DAO facilitates *Salmonella* survival within host immune cells and promotes infection (110). In this section, we will focus on key amino acids that modulate the host response and affect intestinal infections.

Arginine is a key amino acid at the host–pathogen interface. It is synthesized from citrulline in intestinal epithelial cells. Citrulline itself is produced from precursors such as glutamine, glutamate, and proline. In immune cells, arginine is crucial, as it serves as a substrate for inducible nitric oxide synthase (iNOS), which produces nitric oxide (NO). NO is an important antimicrobial molecule and also serves as a signaling mediator that regulates cytokine production and immune response. NO is therefore recognized to play an important role in both innate and acquired immunity (111, 112). Arginine is a substrate not only for iNOS but also for the host’s arginase, which generates ornithine and urea. As these two enzymes compete for the same substrate, the expression and activity of arginase play a critical role in NO generation (113). Some pathogens utilize this competition for arginine to circumvent the detrimental effects of NO. *Helicobacter pylori*, which can persistently colonize the lining of the stomach, constitutively expresses its own arginase, which consumes arginine and thus reduces its availability for NO production by iNOS (114).

Arginine, however, can modulate host immune responses via mechanisms beyond NO production. Alteration in gut arginine availability, whether through dietary supplementation or microbial metabolism, can dampen the inflammatory responses to pathogens (33, 51). For instance, increased arginine availability driven by *C. albicans* arginine production or by modulation of arginine levels by the protist *Tmu* has been associated with decreased proinflammatory cytokine transcript levels (33, 51). However, the consequence of this modulation is context dependent. In the case of *Salmonella* infection, reduced cytokine production and neutrophil recruitment can facilitate pathogen dissemination to peripheral organs (51). In contrast, similar immune modulation has been shown to limit *C. difficile* infection, potentially due to concomitant increase in IFN-γ-mediated responses in the presence of *Tmu* (33).

Another important amino acid in the gut is glutamine, which is often described as a “fuel for the immune system.” This is because immune cells markedly increase their glutamine utilization during infection and inflammation (115). Both the small and large intestines are major sites of glutamine metabolism, consuming large amounts of dietary glutamine (116). Despite this high demand, glutamine concentrations in the intestinal tissue remain relatively low. This is due to the rapid conversion of glutamine to glutamate. Studies have shown that glutamine consumption reduces pro-inflammatory cytokine production in human intestinal mucosa, likely via a post-transcriptional mechanism (115). In mice with dextran sulfate sodium-induced colitis, dietary glutamine attenuates chronic inflammation and promotes expansion of intraepithelial γδ-T cells (117). In addition to its immunomodulatory effects, glutamine plays a critical role in maintaining epithelial barrier integrity. It regulates the expression of tight junction proteins, which are essential for controlling intestinal permeability. In the human colon carcinoma cell line Caco-2, glutamine deprivation leads to a marked reduction in the expression of key tight junction components, including claudin-1, occludin, and ZO-1, accompanied by increased epithelial permeability (10, 118). In line with this, dietary supplementation with glutamine mitigates intestinal inflammation and improves barrier function in broiler chickens infected with *Salmonella enteritidis* (119).

Tryptophan metabolism is another important pathway that links amino acid metabolism to immune regulation. The presence of tryptophan enhances tight junction protein expression in pig intestinal epithelial cells *in vitro* (120). Moreover, microbial catabolism of tryptophan generates a diverse array of indole derivatives, including indole, indole-3-propionate, and indole-3-aldehyde (121). These metabolites play key roles in maintaining intestinal homeostasis by participating in intestinal epithelial cell renewal, barrier integrity, and the differentiation of various immune cell types (122). Indole-3-propionic acid is also produced by *Clostridium sporogenes* and regulates intestinal barrier function via the exogenous pregnane X receptor (123–125). Tryptophan and its downstream metabolites can also bind to the aryl hydrocarbon receptor, which regulates intestinal inflammation, cell proliferation, and differentiation, as well as the transcription and expression of inflammation-related factors (126). Host-mediated tryptophan metabolism further supports appropriate immune responses against pathogens. The enzyme indoleamine 2,3-dioxygenase (IDO) catalyzes the conversion of tryptophan into kynurenine, thereby reducing local tryptophan availability and influencing immune responses. During *C. difficile* infection, IDO expression is upregulated, leading to depletion of the tryptophan pool, which in turn dampens host immune responses against the pathogen and restricts *C. difficile* immunopathology (127).

## CONCLUSION

The complex interactions between the host and diverse microbial communities shape amino acid metabolism in the gut. Microorganisms not only consume dietary amino acids but also generate a wide range of metabolites that influence intestinal homeostasis, immune responses, metabolic regulation, and gut–brain communication. Therefore, any disruption in microbiota-driven amino acid metabolism can contribute to the development and progression of diseases. In addition, these metabolic interactions also affect enteric infections. Enteric pathogens may compete for limited amino acid resources or sense amino acids as signals that regulate their virulence. Conversely, pathogen-induced changes in amino acid metabolism can further disrupt epithelial barrier integrity and immune responses. Because many enteric pathogens rely on amino acids as nutrients, strategies that manipulate microbial amino acid metabolism or introduce microbes that modulate amino acid availability could provide new approaches to enhance colonization resistance. Such approaches may be particularly valuable for combating multidrug-resistant pathogens, including *A. baumannii*, where conventional antibiotic options are limited.

Amino acids supplements are frequently prescribed or recommended to promote growth and health. For example, arginine is a popular dietary supplement in the United States for cardiovascular diseases and exercise tolerance, and has been suggested for the treatment of dementia, hypertension, cancer, male infertility, diabetes, and obesity (128–136). However, using arginine might modulate the susceptibility to enteric infections such as *Salmonella* and *C. difficile*, suggesting the need for more informed dietary supplementation strategies. Studies have shown that amino acid supplementation can alter the diversity and composition of microbes in the gut. It is interesting to note that some effects of amino acid supplementation were observed in the large intestine, even though free amino acids are thought to be absorbed primarily in the small intestine. This may happen because very high levels of amino acids temporarily exceed the small intestine’s ability to absorb them (19). Therefore, despite significant advances, many aspects of amino acid metabolism in the gut are unknown. This is particularly true for non-bacterial members of the microbiome. Fungal components of the microbiome were initially thought to be metabolically silent as the introduction of a defined fungal community in germ-free mice resulted in only minimal changes to the overall gut metabolome (54). However, a recent study showed that gut fungi can produce and release the amino acid arginine (51), indicating that the role of non-bacterial microbiota members in shaping intestinal metabolic landscape might be greater than previously appreciated.

Altered amino acid levels have been associated with conditions such as obesity, diabetes and cancer. While bacterial communities are known to shape amino acid metabolism in the gut and influence metabolic disorders such as diabetes (16), the contributions of fungi, archaea, and protists to these processes remain unexplored. Furthermore, little is known about whether non-bacterial microbiota interact with bacterial communities in the gut via amino acid exchange or competition. It is also unclear how these interactions change during infection and disease. Addressing these questions will provide a comprehensive understanding of how microbial metabolism shapes gut metabolism and host physiology.
